# Establishing RNA virus resistance in plants by harnessing CRISPR immune system

**DOI:** 10.1111/pbi.12881

**Published:** 2018-02-14

**Authors:** Tong Zhang, Qiufeng Zheng, Xin Yi, Hong An, Yaling Zhao, Siqi Ma, Guohui Zhou

**Affiliations:** ^1^ Guangdong Province Key Laboratory of Microbial Signals and Disease Control College of Agriculture South China Agricultural University Guangzhou Guangdong China; ^2^ Key Laboratory of Pesticide and Chemical Biology Ministry of Education College of Agriculture South China Agricultural University Guangzhou Guangdong China; ^3^ Division of Biological Sciences University of Missouri Columbia MO USA

**Keywords:** CRISPR, *Francisella novicida*, direct targeting, RNA virus, resistance

## Abstract

Recently, CRISPR‐Cas (clustered, regularly interspaced short palindromic repeats–CRISPR‐associated proteins) system has been used to produce plants resistant to DNA virus infections. However, there is no RNA virus control method in plants that uses CRISPR‐Cas system to target the viral genome directly. Here, we reprogrammed the CRISPR‐Cas9 system from *Francisella novicida* to confer molecular immunity against RNA viruses in *Nicotiana benthamiana* and *Arabidopsis* plants. Plants expressing FnCas9 and sgRNA specific for the cucumber mosaic virus (CMV) or tobacco mosaic virus (TMV) exhibited significantly attenuated virus infection symptoms and reduced viral RNA accumulation. Furthermore, in the transgenic virus‐targeting plants, the resistance was inheritable and the progenies showed significantly less virus accumulation. These data reveal that the CRISPR/Cas9 system can be used to produce plant that stable resistant to RNA viruses, thereby broadening the use of such technology for virus control in agricultural field.

## Introduction

Viruses are mobile genetic elements that rely on infecting cellular organisms (eukaryotes or prokaryotes) for replication and proliferation. Viral invasions often reduce the fitness of their host, sometimes leading to host death (Boualem *et al*., 2016; Laliberté and Sanfaçon, 2010). Plant viruses represent a major burden on the global agricultural industry. Genetic engineering has been widely used to combat invading plant viruses in economically important crops. For example, RNA interference (RNAi) is an effective technology that has been used to control plant viruses since the late twentieth century (Ding and Voinnet, 2007; Lindbo and Dougherty, 2005; Qu, 2010). For example, transgenic papaya expressing the coat protein or replicate protein from *Papaya ringspot virus* (PRSV) has been approved for commercial production worldwide, in which RNAi plays the determinant role against PRSV (Ye and Li, 2010). RNAi offers a very promising approach for generating sequence‐specific resistance against multiple viruses in transgenic plants and can target endogenous or exogenous mRNAs transcripts (Ding, 2010; Lindbo and Dougherty, 2005; Qu, 2010). However, through evolution, plant viruses have developed a range of counter‐defensive measures against RNA silencing, one of which is the expression of suppressor proteins that inhibit distinct steps of the silencing pathways (Burgyán and Havelda, 2011; Voinnet, 2005).

The clustered, regularly interspaced short palindromic repeats (CRISPR)/CRISPR‐associated (Cas) 9 (CRISPR/Cas9) systems comprises sequence‐specific RNA‐directed endonuclease complexes that bind and cleave nucleic acids (Bhaya *et al*., 2011). In recent years, besides using its powerful capability for genome editing (Cong *et al*., 2013; Mali *et al*., 2013), the CRISPR/Cas9 machinery has been exploited to combat eukaryotic viruses (Price *et al*., 2016; Scheben *et al*., 2017; Zaidi *et al*., 2016), in particular, it has been successfully applied in inhibiting DNA virus infection in plants by targeting the viral genome (Ali *et al*., 2015; Baltes *et al*., 2015; Ji *et al*., 2015). This strategy has the advantage that the eukaryotic viruses have not evolved to possess the ability to counter this immune defence. However, because of the limitations of type II CRISPR/Cas systems, Cas9 has been reported to target and cleave dsDNA only (Doudna and Charpentier, 2014). For RNA viruses, which cause more serious losses in crops and enormously damage to agricultural production, no effective and valid method to control them using CRISPR/Cas9 has been reported in plants (Zaidi *et al*., 2016). Encouragingly, some Cas protein variants, like the Cas9 from *Francisella novicida* (FnCas9) and the type VI‐A CRISPR/Cas effector from *Leptotrichia shahii* (LshCas13a) or *Leptotrichia wadei* (LwaCas13a), have been reported to target RNA *in vivo* (Abudayyeh *et al*., 2016, 2017; Sampson *et al*., 2013). These findings offered us an excellent chance to directly target RNA viruses within eukaryotes.

Here, we reprogrammed and expressed the FnCas9 and its RNA‐targeting guide RNA in plants. To our knowledge, this is the first report of a method targeting the viral RNA to control plant viruses. The cucumber mosaic virus (CMV) and tobacco mosaic virus (TMV), both are positive‐sense RNA plant virus that can infect many species (Palukaitis and García‐Arenal, 2003), were used to test the virus inhibition efficiency of the CRISPR/Cas9 system from *F. novicida in planta*. Given the proven flexibility of this system to confer resistance to RNA viruses, we believe that the protocols and procedures outlined here could be optimized and modified for using to control many other agricultural plant viruses.

## Results

### *Francisella novicida* CRISPR/Cas9 expression in plants and target sites selection

**Figure 1:**
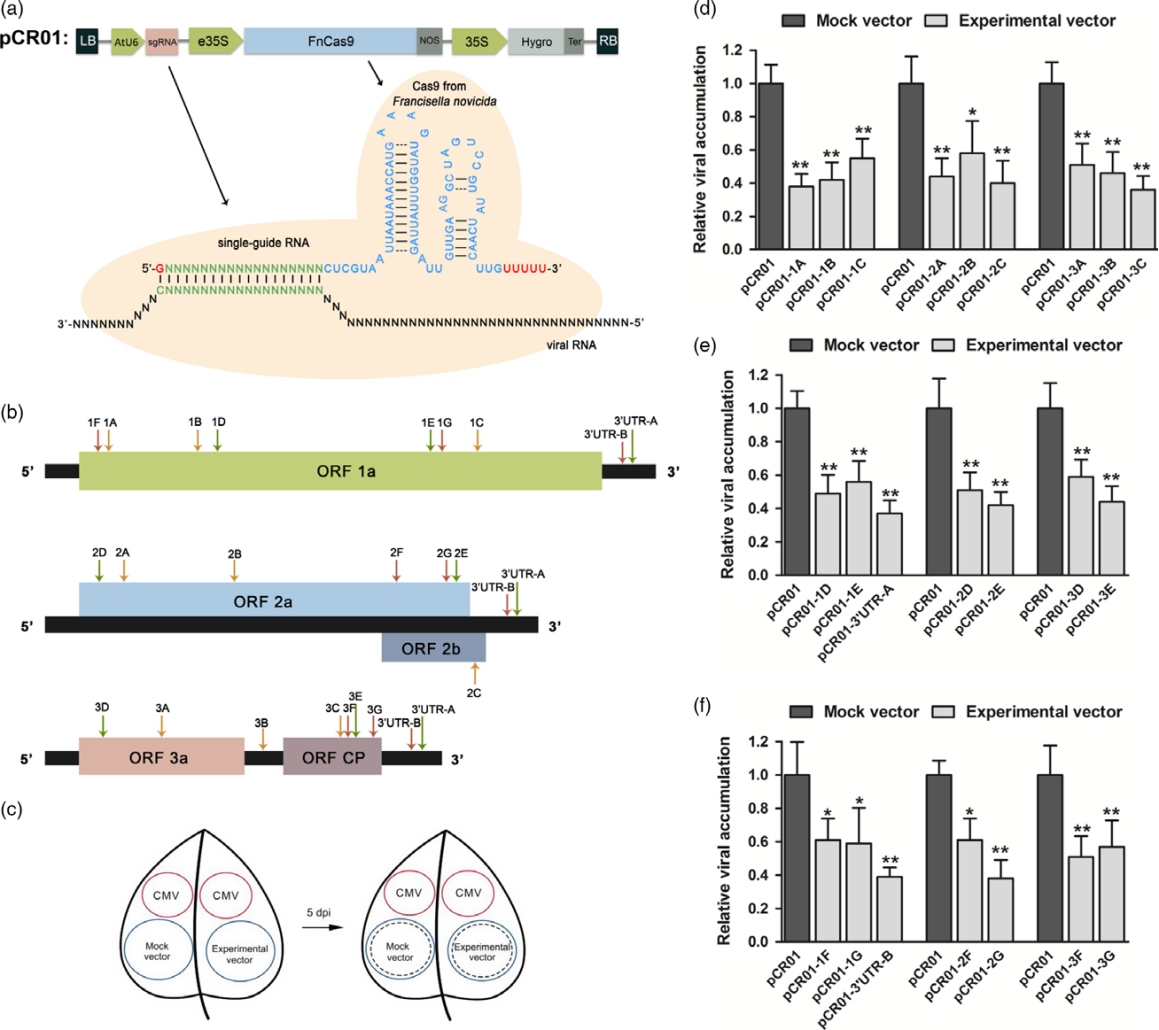
Expressing the *Francisella novicida* CRISPR‐Cas9 system in plants and screening viral target sites. (a) Schematic illustrating the engineered *F. novicida* CRISPR‐Cas9 system. (b) CMV genome structure and design of the sgRNA target sites. The schematic shows the three segments of the CMV genome and the positions of the five open reading frames. Three types of arrows on the genome indicate the positions of the 22 sgRNA target sites: type I, orange arrow, NGG PAM in the viral genome complementary sense; type II, green arrow, NGG PAM in the viral genome sense; type III, red arrow, PAM with no common features. (c) Screening of suitable sgRNA target sites for inhibition of virus infection. Leaves from a 25‐day‐old *Nicotiana benthamiana* plants were marked with four circular regions. The CMV constructs were injected into the top circular regions (red), and one of the experimental vectors and the control vector were injected separately into the bottom circular regions (blue). Five days after injection, the RNA in the bottom regions (circles in dashed lines) was extracted and viral accumulation was measured by RT‐qPCR. (d–f) CMV accumulation in the screening samples for the three types of sgRNA target sites. Error bars represent s.d.; asterisks indicate significance: *P < 0.05, **P < 0.01. Data are representative of three biological replicates.

In this study, we constructed a pCambia1300‐derived vector pCR01 which contained a codon‐optimized *F. novicida* Cas9 (FnCas9) driven by an enhanced 35S promoter and a single‐guide RNA (sgRNA) driven by an AtU6 promoter (Figure 1a).

The CMV has a tripartite genome of positive‐sense, single‐stranded RNAs of about 8600 nucleotides (nt) with five open reading frames (ORFs), and the 3′ untranslated regions (3′ UTRs) of the three genomes present very high nucleotide sequence similarity (Figure 1b; Palukaitis and García‐Arenal, 2003). To identify highly efficient sgRNA–FnCas9 target sites, we selected 23 candidate sites including two common ones located in the 3′ UTRs (Figure 1b and Table S1). For DNA targeting, the Cas9 protein requires a short sequence adjacent to the targeted region, called a proto‐spacer adjacent motif (PAM) (Doudna and Charpentier, 2014). To determine whether the PAM was necessary for RNA targeting, three types of PAM were included in these 23 target sites, NGG in the viral genome complementary sense, NGG in the viral genome sense and a PAM with no common features. We synthesized complementary oligonucleotides based on the target sequences and inserted them into the pCR01 vector to create 23 corresponding pCR01‐sgRNA vectors (Table S1).

### Screening of the sgRNA–FnCas9 target sites

To screen the inhibitory effect of the pCR01‐sgRNAs on CMV replication, we developed a transient assay in which we simultaneously injected *Agrobacteria* containing a given experimental vector and the control vector together with CMV agro‐infectious clones into individual 25‐day‐old *N. benthamiana* leaves. The CMV agro‐infectious clones were injected into the top part of the leaf and the experimental vector and the control vector were injected into separate areas of the bottom part of the leaf (Figure 1c). Five days after injection, leaf discs from the areas in the bottom of the leaves injected with the experimental or control vector were harvested. Quantification of virus RNA by RT‐qPCR showed that all the sgRNA–FnCas9 constructs inhibited virus accumulation in the injected regions (Figure 1d–f). Compared with the control vector, all the constructs reduced the viral RNA by over 40%, and five constructs reduced it by over 60%, regardless of whether the PAM was NGG in the viral genome complementary sense (Figure 1d), NGG in the viral genome sense(Figure 1e), or had no common features (Figure 1f). These results suggested that we had established an efficient system for suitable sgRNA target sites screening, and demonstrated that FnCas9‐mediated inhibition of CMV infection was independent of the sequences adjacent to the targeted region.

### Establishing resistance in tobacco

**Figure 2:**
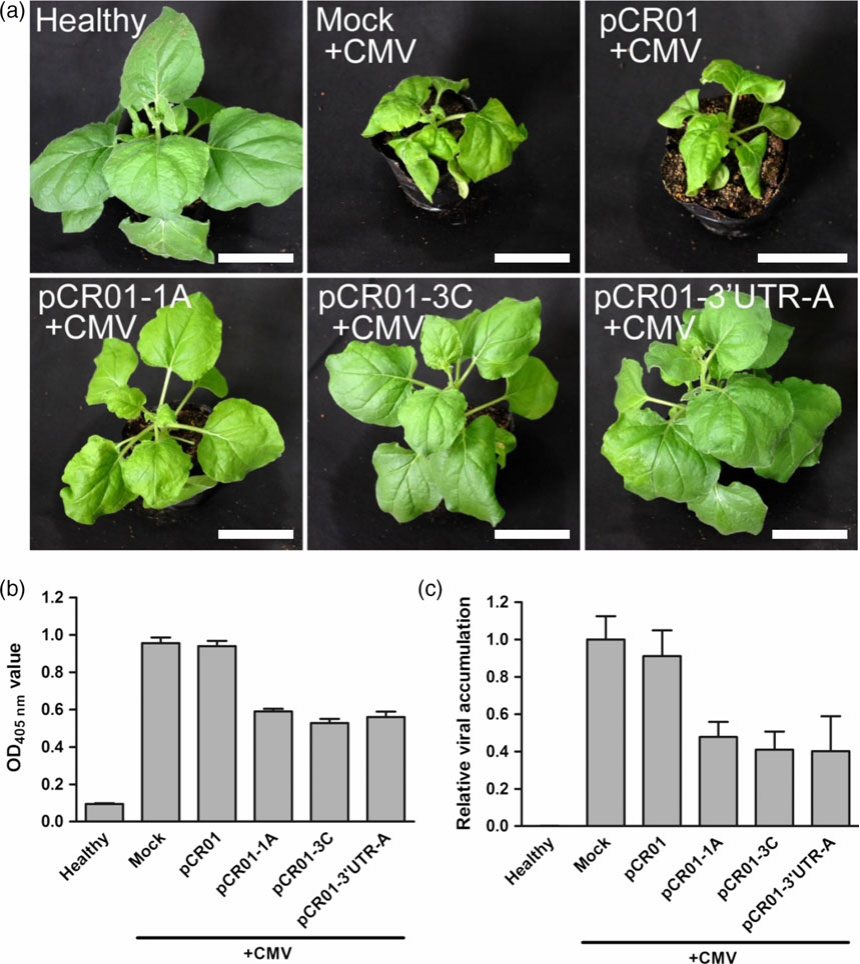
Transient expression of the *Francisella novicida* CRISPR‐Cas9 system in *Nicotiana benthamiana* conferred resistance to CMV. (a) Symptoms of *N. benthamiana* plants transiently expressing pCR01‐sgRNAs and control vectors after CMV inoculation. Scale bars = 5 cm. (b, c) CMV accumulation in *N. benthamiana* plants was assessed by ELISA (b) and RT‐qPCR (c). Error bars represent SD. Data were representative of three biological replicates.

The sgRNAs targeting sites, 1A, 3C and 3′UTR‐A, were selected for further experiments because they showed high efficiency in the screening assay (Figure 1d–f). The selected vectors, pCR01‐1A, pCR01‐3C and pCR01‐3′UTR‐A, together with the pCR01 vector as a control were transiently expressed in *N. benthamiana* by agroinoculation, and 1 day later, two leaves were inoculated with CMV (Figure S1). After 2 weeks, typical CMV symptoms, such as leaf shrinkage, were observed on infected control plants (Figure 2a). However, in plants inoculated with the highly efficient sgRNAs, pCR01‐1A, pCR01‐3C and pCR01‐3′UTR‐A, no severe leaf shrinkage symptoms were observed in either infected or systemic leaves. Quantification of CMV titre by enzyme‐linked immunosorbent assay (ELISA) showed that virus accumulation in plants inoculated with the three pCR01‐sgRNA vectors was reduced by 40–50%, compared with mock‐inoculated or Cas9 only vector‐inoculated plants (Figure 2b). Viral genomic RNA was also quantified by RT‐qPCR, and the results confirmed that pCR01‐1A, pCR01‐3C and pCR01‐3′UTR‐A inhibited CMV accumulation in inoculated plants (Figure 2c).

**Figure 3:**
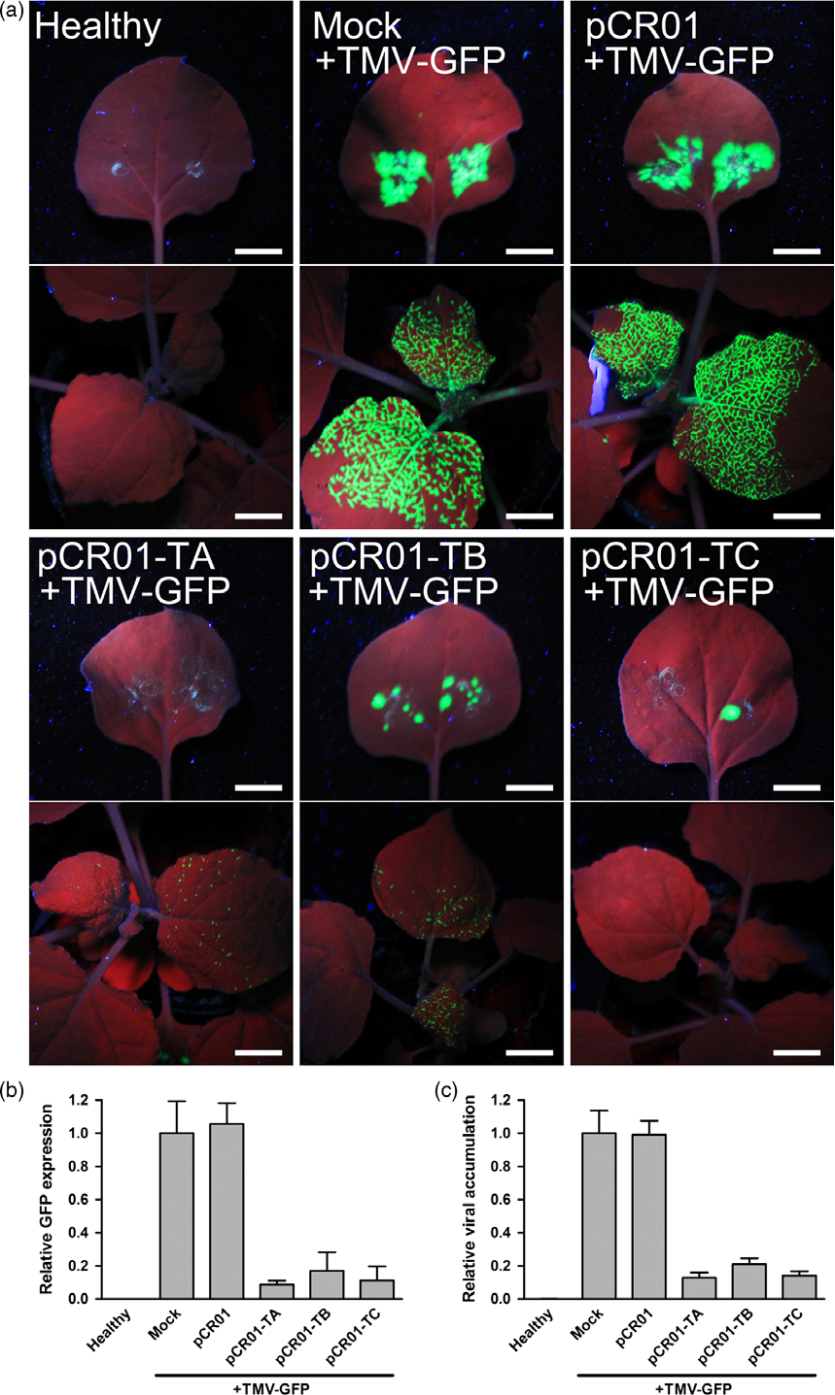
Transient expression of the *Francisella novicida* CRISPR‐Cas9 system in *Nicotiana benthamiana* conferred resistance to TMV. (a) GFP fluorescence of *N. benthamiana* plants transiently expressing pCR01‐sgRNAs and control vectors after TMV‐GFP inoculation. The inoculated leaves (upper) and systemic leaves (lower) of each inoculating combination were taken pictures separately. Scale bars = 1 cm. (b, c) GFP expression level (b) and TMV accumulation (c) in *N. benthamiana* plants were assessed RT‐qPCR. Error bars represent SD. Data were representative of three biological replicates.

In order to visualize the inhibition of virus infection and test the resistance to other RNA virus, the agro‐infectious clone of TMV‐GFP was employed in the assay, in which the fluorescence protein was inserted and would be expressed by TMV infection (Figure S1). Three sgRNAs were designed to targeting the TMV coding region and inserted into pCR01 vector to create pCR01‐TA, pCR01‐TB and pCR01‐TC (Table S1). At 1 week postinoculation, bright green fluorescence was observed in both inoculation sites and systemic leaves of the control plants (Figure 3a, mock or pCR01 pre‐inoculated). In pCR01‐TA, pCR01‐TB or pCR01‐TC pre‐inoculated tobacco plants, the green fluorescence was obviously weaker in both inoculation sites and systemic leaves (Figure 3a), which reflects that the TMV infection was significantly attenuated by the CRISPR/Cas9 system. Quantification of the GFP expression level (Figure 3b) and TMV titre (Figure 3c) by RT‐qPCR further confirmed that the TMV‐GFP levels in CRISPR targeted plants were significantly decreased.

Therefore, these results have proved that the *F. novicida* CRISPR/Cas9 system guided by sequence‐specific sgRNA could target the RNA virus, and suppress its infection in plants.

### FnCas9 could bind viral RNA by sgRNA directing

**Figure 4:**
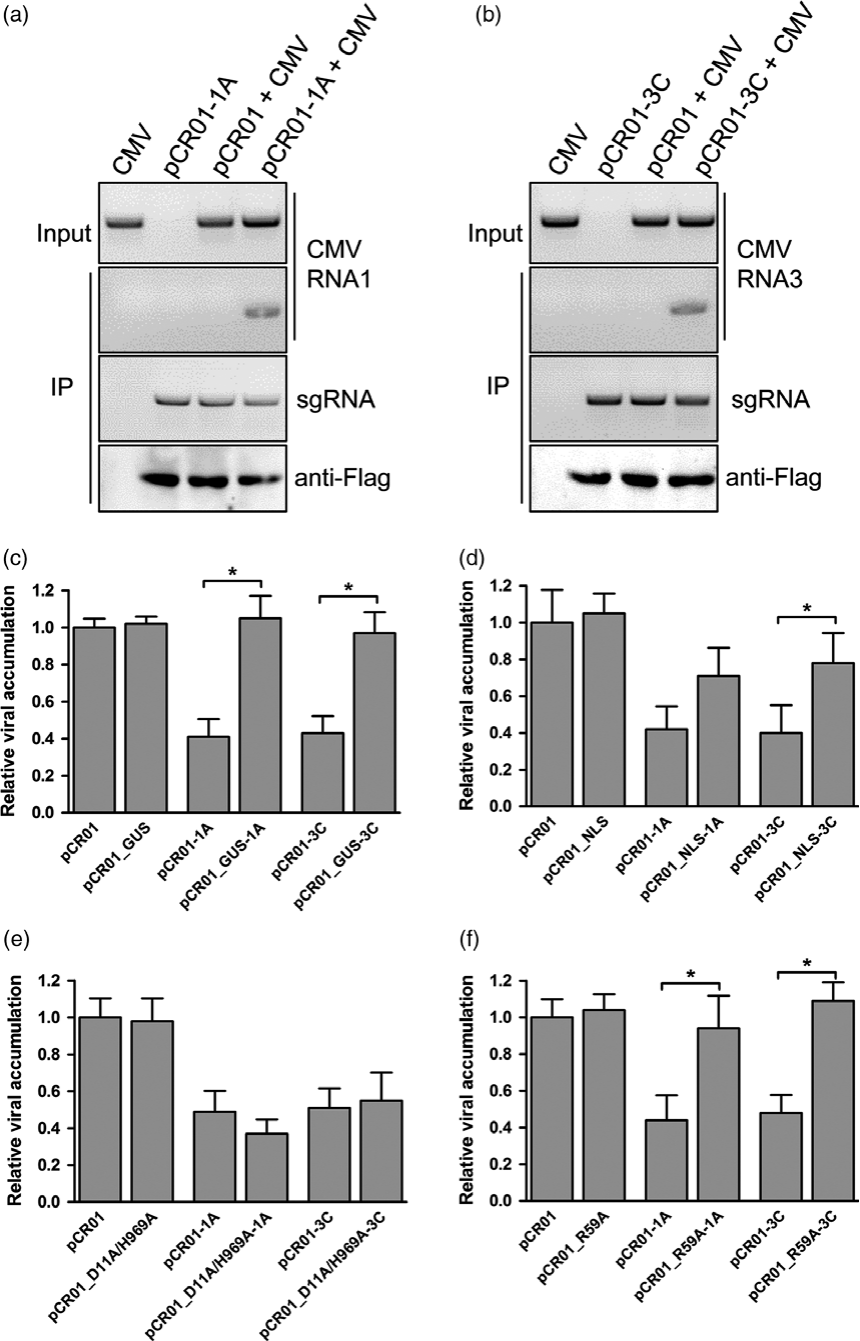
FnCas9 targets CMV RNA by directly binding. (a, b) N. *benthamiana* plants transiently expressing FnCas9 with sgRNAs targeting the 1A site (a) or 3C site (b) were co‐inoculated with CMV. At five days postinfection, lysates were immunoprecipitated with anti‐FLAG. The coprecipitating RNA was purified and analyzed by RT‐PCR to detect the corresponding viral RNA. A western blot of FnCas9 was performed to confirm the FnCas9 proteins were successfully expressed and immunoprecipitated. The results from one out of two independent experiments are displayed. (c‐f) Several chimeric pCR01vectors with GUS replaced FnCas9 (c), FnCas9 attached to a NLS (d), alanine double point mutations in the RuvC and HNH domain (D11A/H969A) (e), and alanine point mutation in the ARM (R59A) (f) were tested for the virus inhibition efficiency by transiently expressing them with sgRNA targeting 1A or 3C sites in the CMV genome. Virus accumulation was assessed by RT‐qPCR. Error bars represent s.d.; asterisks indicate significance: * P <0.01. Data are representative of three biological replicates.

Co‐immunoprecipitation was performed to determine whether sgRNA–FnCas9 was directly associated with CMV genomic RNA. Because the FnCas9 protein expressed by pCR01 was fused with a 3×FLAG tag at its N‐terminus (Appendix S1), FnCas9 was immunoprecipitated from pCR01‐sgRNA and CMV inoculated leaves, the RNA associated with FnCas9 was purified, and RT‐PCR was performed for the sgRNA and CMV. The Western blot for 3×FLAG‐FnCas9 suggested that the FnCas9 proteins were successfully expressed and immunoprecipitated, and the sgRNAs were also precipitated by binding to the FnCas9 (Figure 4a,b). CMV genomic segment 1 and 3 were detectable in plants co‐inoculated with pCR01‐1A (Figure 4a) or pCR01‐3C (Figure 4b), respectively, and the CMV genomic RNA were not present in the nonspecific sgRNA‐inoculated sample. Thus, FnCas9 associated with the viral RNA targeted by sequence‐specific sgRNA within plants.

### The RNA‐binding, not cleavage, capacity of FnCas9 is essential for virus inhibition

To determine how FnCas9 inhibited CMV infection in the plant, we tested several chimeric pCR01 vectors using our screening system. First, to exclude the possibility that the sgRNA bind to the viral genome and inhibit the infection without the help of FnCas9, a GUS gene was substituted for the FnCas9 to produce pCR01_GUS (Figure S2). When targeted to the CMV 1A or 3C sites, both pCR01_GUS‐1A and pCR01_GUS‐3C lost the ability to suppress the CMV infection as pCR01‐1A and pCR01‐3C (Figure 4c). Then, a nuclear localization signal (NLS) was added to both ends of the FnCas9 protein, generating pCR01_NLS, to limit the *F. novicida* CRISPR/Cas9 system to the nuclei of plant cells (Figure S2), the results showed that the nuclear localization of FnCas9 impaired its repression of CMV infection (Figure 4d). Next, two alanine point mutations in the conserved RuvC and HNH active sites of FnCas9 (D11A and H969A; Sampson *et al*., 2013) were generated to test whether the endonucleolytic activity was involved in the repression of virus infection (Figure S2). Interestingly, both wild‐type and D11A/H969A FnCas9 showed similar inhibitory effects on CMV infection (Figure 4e). Lastly, to test whether the RNA‐binding activity was essential for the repression of virus infection, another alanine point mutation was generated in the RNA‐binding arginine‐rich motif (ARM) of FnCas9 (R59A; Figure S2). Both pCR01_R59A‐1A and pCR01_R59A‐3C abrogated the repression of CMV infection compared with pCR01‐1A and pCR01‐3C (Figure 4f). Taking these results together, we concluded that the ARM region, not the cleavage sites, of the FnCas9 was important for inhibiting CMV infection in our system.

### Establishing stable resistance in *Arabidopsis*

**Figure 5:**
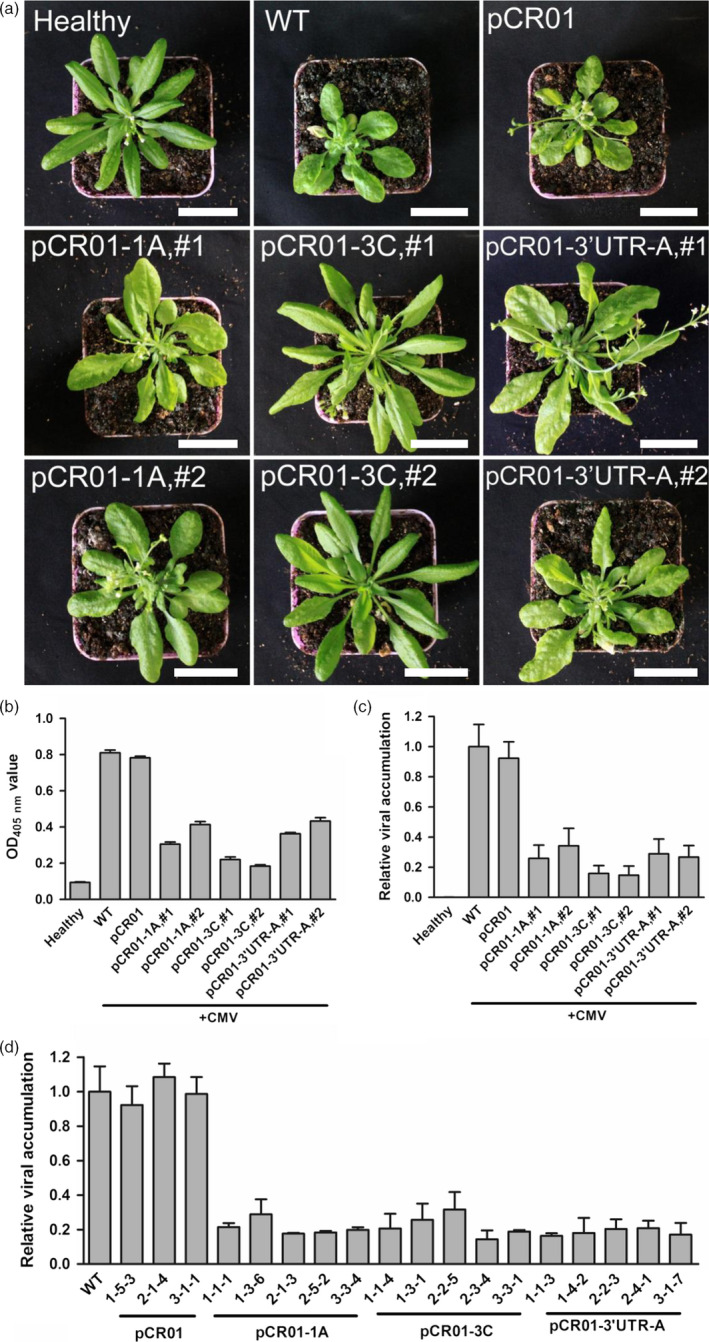
Transgenic *Arabidopsis* plants established resistance to CMV. (a) The symptoms of T2 transgenic *Arabidopsis* after CMV inoculation. Two T2 homozygous lines for each transgenic pCR01‐sgRNA constructs were chosen for virus attacks. Scale bars = 3 cm. (b, c) Virus accumulation in T2 transgenic *Arabidopsis* plants was assessed by ELISA (b) and RT‐qPCR (c). (d) T6 transgenic lines were randomly selected and attacked by CMV, the virus accumulation was assessed by RT‐qPCR. Error bars represent SD. Data were representative of three biological replicates.

Finally, we examined the ability of sgRNA–FnCas9 to inhibit CMV infection in stable transgenic plants. Using *Agrobacterium*‐mediated transformation, pCR01‐1A, pCR01‐3C, pCR01‐3′UTR‐A, alone with the control vector pCR01 were introduced into *Arabidopsis*. T2 transgenic homozygous lines for each construct, along with control wild‐type *Arabidopsis*, were selected and infected with CMV by mechanical inoculation. Two weeks later, severe symptoms were observed in the control plants, including leaf deformity and delayed growth (Figure 5a). In the transgenic lines, most plants showed mild symptoms, and the two pCR01‐3C lines in particular had no obvious symptoms (Figure 5a). Quantification of virus accumulation by ELISA (Figure 5b) and RT‐qPCR (Figure 5c) showed that CMV infection was indeed inhibited in these transgenic plants. To test the inheritability of the resistance, we continuously harvest the progenies until T6 generation, and randomly selected several lines to attack by CMV. Inspiringly, all the T6 transgenic plants we tested showed stable resistance to CMV (Figure 5d). Our results demonstrated that overexpressing of sgRNA–FnCas9 specifically targeting sequences in the viral genome was an effective way to generate RNA virus‐resistant plants.

## Discussion

The CRISPR/Cas9 system has been developed as a powerful tool for genome editing in the past few years (Doudna and Charpentier, 2014). The most widely used CRISPR/Cas9 system is derived from *Streptococcus pyogenes* and a synthetic sgRNA that directs the Cas9 endonuclease to a target sequence complementary to the 20 nucleotides preceding the PAM NGG, which is required for Cas9 activity (Cong *et al*., 2013; Mali *et al*., 2013). In 2013, it was reported the Cas9 from *F. novicida* (FnCas9) could target RNA, which presented the possibility of developing RNA virus resistance using genome editing technology (Sampson *et al*., 2013). Price *et al*. (2015) used this *F. novicida* CRISPR/Cas9 system to target the genome of a positive‐sense single‐stranded RNA (+ssRNA) virus, the hepatitis C virus (HCV), within hepatoma cell lines. Transient expression of Cas9 and sgRNA complexes targeted to either the 5′ or 3′ untranslated regions of the HCV genome resulted in a 50–60% reduction in viral protein expression relative to the control. In our study, the *F. novicida* CRISPR/Cas9 system was expressed both transiently and stably in plants, and the plants were inoculated with CMV or TMV to evaluate the feasibility of using CRISPR/Cas9‐mediated interference against RNA viruses. We selected 23 target sites for CMV and three sites for TMV, all of them could inhibit virus accumulation by 40–80% compared with the nontargeting control, showing that the *F. novicida* CRISPR/Cas9 system has similar or even better virus interference efficiency in plants as in mammalian cells.

The PAM, an NGG trinucleotide, is essential for Cas9 to cleave DNA. In the 25 target sites we screened, three types of PAM were included, NGG in the viral genome complementary sense, NGG in the viral genome sense, and a PAM with no common features. Regardless of which type of PAM was present, viral infection was inhibited. Furthermore, the cytosolic localization of FnCas9 was important for inhibition of viral infection (Figure 4a), in contrast to DNA targeting by Cas9, which relies on nuclear localization (Hsu *et al*., 2013). This cytosolic RNA‐targeting feature of FnCas9 would potentially limit off‐target effects of FnCas9 on the host DNA.

By generating point mutations, we found that the endonuclease activity of FnCas9 was not required for FnCas9‐mediated inhibition of CMV (Figure 5b), while the RNA‐binding activity was indispensable (Figure 5c). These data suggest that inhibition was most likely due to the blocking of viral genome replication and protein translation by FnCas9 binding to the viral genome, rather than viral genome cleavage, and our data showed that our system is FnCas9 dependent (Figure 4c), which demonstrated that the inhibitory effect is not due dsRNA (sgRNA‐viral RNA duplex) triggered RNAi effect. These results were consistent with FnCas9 inhibition of HCV in cell lines (Price *et al*., 2015), which means the application of FnCas9 to target RNA can be more accurately defined as CRISPR interference (CRISPRi), in which catalytically inactive Cas9 proteins are programmed to dampen gene expression (Larson *et al*., 2013). Compared with engineered CRISPR/Cas9‐based resistance against DNA viruses (Ali *et al*., 2015; Baltes *et al*., 2015; Ji *et al*., 2015), which created mutations in the viral genome and resulted in the generation of viral variants capable of escaping resistance (Ali *et al*., 2016), the CRISPRi‐like FnCas9 system targeting RNA viruses has its advantages. FnCas9 bound but did not cleave the viral genome, and this characteristic could limit the generation of mutated viral variants capable of escape, which is essential for developing durable resistance strategies for long‐term virus control.

In the past 20 years, RNAi has been a very popular strategy for virus control in mammals and plants (Ding, 2010; Ding and Voinnet, 2007). However, through long‐term co‐evolution, eukaryotic viruses have developed methods of circumventing RNAi, such as by encoding an RNAi suppressor, which leads to a crackdown of interference efficiency (Burgyán and Havelda, 2011; Voinnet, 2005). Conversely, the CRISPR/Cas9 is an adaptive molecular immunity system used by prokaryotes (Garneau *et al*., 2010; Horvath and Barrangou, 2010; Marraffini and Sontheimer, 2010), so eukaryotic viruses have not evolved in the presence of Cas9, meaning they are unlikely to have Cas9 evasion strategies. Our results demonstrated that the resistance acquired from CRISPR/Cas9 was quite stable, which could be inherited at least until the T6 generation (Figure 5d). Therefore, the CRISPR/Cas9 system has a natural advantage over RNAi, being able to interfere with viral infection without the eukaryotic virus escaping. One important issue should not be forgotten is that our system is also sequence‐based and during the RNA viruses evolving, there might have some mutations in the target sites which might lead the virus escaping from the CRISPR/Cas9‐mediated resistance. There are two solutions can be effectively reduce this risk, one is positioning the target sites in relatively conservative regions of the virus genome where has much lower probability of mutation, and the other one is transforming more than one sgRNA which makes our system has multiple targeting sites of the virus and can tolerate more virus variants.

Besides the FnCas9 we used, other RNA‐targeting Cas proteins have been reported. O'Connell *et al*. (2014) reported that purified Cas9 from *S. pyogenes* (SpCas9), along with its guide RNA, could bind and cleave single‐stranded RNA in the presence of an exogenous PAM oligonucleotide, and newly identified a Class 2 type VI CRISPR‐Cas system endonuclease LshCas13a and LwaCas13a, as an RNA‐targeting CRISPR effector (Abudayyeh *et al*., 2016, 2017). These findings provide us with more options for developing antiviral strategies based on CRISPR, especially the Cas13a, which has the capacity to cleave RNA and can be used to generate RNA virus resistance plants by cutting the viral genome (Price *et al*., 2016; Zaidi *et al*., 2016). Over time, the two strategies derived from FnCas9 and C2c2, binding and cleaving, respectively, could be compared to determine which is more efficient at inhibiting RNA virus infection.

In summary, by expressing the *F. novicida* Cas9 protein with a sgRNA in the same vector, we successfully established interference against RNA viruses in plants. Our data demonstrate that the prokaryotic immune system CRISPR/Cas9 can be successfully adapted for eukaryotic intracellular defence against invading RNA viruses, and that this immune system could be used in other host plants to confer resistance to any sequenced RNA virus.

## Materials and methods

### Plants and virus

*Arabidopsis thaliana* ecotype Col‐0 and *Nicotiana benthamiana* plants used in this study were grown as previously described (Zhang *et al*., 2015, 2016). Agro‐infectious clones of TMV‐GFP (Shivprasad *et al*., 1999) and CMV (Fny strain) (Liao *et al*., 2015) and were kindly provided by Prof. Xueping Zhou (Zhejiang University) and Dr. Zhouhang Gu (Zhejiang Sci‐Tech University), respectively.

### Vector construction

An *F. novicida* Cas9 (FnCas9) gene sequence including a 3×Flag tag fused to the N‐terminus was designed with codon optimization for plants (Appendix S1). This gene and the 90‐bp chimeric sgRNA containing two *Bsa*I digestion sites were synthesized by GeneScript (Nanjing, China). The *FnCas9* gene was linked to an enhanced 35S promoter and NOS terminator, while the sgRNA was linked to the *Arabidopsis* U6 promoter. These two expression cassettes were inserted into a binary vector derived from pCAMBIA1300 (Cambia, Canberra, ACT, Australia), in which the original *Bsa*I site was removed. The final vector was named pCR01 and was used for transient and stable expression of the *F. novicida* CRISPR/Cas9 system in dicot plants.

NLSs were attached to both ends of FnCas9 to generate FnCas9_NLS. Point‐mutated *FnCas9* genes were constructed using a Fast Mutagenesis Kit (Vazyme, China) to generate FnCas9_D11A/H969A and FnCas9_R59A. All the three modified *FnCas9* genes were substituted for the FnCas9 in pCR01 to produce pCR01_NLS, pCR01_D11A/H969A and pCR01_R59A, respectively. The β*‐Glucuronidase* (GUS) gene was substituted for the FnCas9 to produce pCR01_GUS. The pCR01 and its variant vectors were digested by BsaI and ligated with the synthesized sgRNA oligos (Table S1). All constructs were authenticated by sequencing prior to introduction into *Agrobacterium tumefaciens* strain EHA105.

### Tobacco transient expression

EHA105 strains containing all pCR01–sgRNA vectors or the pCR01 vector were grown in YEP medium with kanamycin and rifampicin overnight. The agrobacteria were harvested by centrifugation and diluted in inoculation buffer (10 mm MES pH5.6, 10 mm MgCl_2_, 100 μm acetosyringone) to a final OD_600 nm_ of 1.0. Four true leaves of 4‐week‐old *N. benthamiana* plants were chosen and injected with the agrobacteria. One day later, CMV or TMV‐GFP infectious clones with a final OD_600 nm_ of 0.6 were injected into the bottom two previously infected leaves.

### Transgenic *Arabidopsis* generation

*Agrobacterium tumefaciens* harbouring the selected constructs was transformed into *Arabidopsis* using a floral dip method. Seeds harvested from the resulting plants (T1) were screened for resistance to hygromycin (20 μg/mL). The surviving seedlings were tested for transgenic expression of FnCas9 by RT‐qPCR. Two selected lines of each construct were further propagated for CMV attacking by mechanical inoculation.

### RNA extraction and RT‐qPCR

The injected or systemic leaves were picked, and the RNA was extracted with TRIzol (Invitrogen, Carlsbad, CA). For viral RNA quantification, one‐step RT‐qPCRs were carried out in a CFX96 Touch™ real‐time PCR detection system (Bio‐Rad, Hercules, CA) using HiScript II Q RT SuperMix (Vazyme, Jiangsu, China). For FnCas9 transgenic and GFP expression measurement, cDNA was synthesized from total RNA using the oligo(dT) primer and reverse transcriptase (Takara). RT‐qPCRs were carried out in the same instrument using SYBR^®^ Premix Ex Taq™ II (Takara, China). Three replicates were conducted for each treatment, and three technical RT‐qPCR replicates were analysed for each biological replicate. The tobacco *PP2A* or *Arabidopsis ACTIN8* gene was used as reference. Primers for RT–qPCRs are shown in Table S2.

### TAS‐ELISA

The CMV titre was also quantified using the triple antibody sandwich enzyme‐linked immunosorbent assay (TAS‐ELISA) following the standard procedures. Systemic leaves (100 mg) of infected tobacco or *Arabidopsis* were isolated for analysis, and three replicates were conducted for each treatment.

### Immunoprecipitation

Anti‐Flag immunoprecipitation was performed according to the manufacturer's instructions (Sigma‐Aldrich, USA) from *N. benthamiana* leaves inoculated with pCR01‐sgRNA vectors and infected with CMV as indicated. The immunoprecipitated proteins were confirmed by Western blotting. Total RNA was extracted from the precipitated fraction using an RNeasy Mini Kit (Qiagen, Valencia, CA) according to the manufacturer's instructions, and the CMV genomic RNA and sgRNAs was further detected by RT‐PCR using a HiScript II One Step RT‐PCR Kit (Vazyme).

## Author contributions

T.Z. and G.Z conceived the project. T.Z., H.A. and G.Z. designed the research. T.Z., Q.Z., X.Y., Y.Z. and S.M. performed the research. T.Z., X.Y., H.A. and G.Z. analysed the data. T.Z. and G.Z. wrote the article with contributions from all the authors.

## Competing interests

T.Z. and G.Z. have filed a patent application in China (priority filing with serial number 201710182462.3).

## Supporting information


**Figure S1** Transient transformation of the sgRNA–FnCas9 system to develop CMV or TMV resistance in *N. benthamiana*.
**Figure S2** Structure of pCR01 and its variants used in the transient assay.
**Table S1** sgRNA target site IDs, sequences and oligonucleotides for cloning into pCR01.
**Table S2** Primers used in this study.
**Appendix S1** Sequence of the sgRNA and FnCas9 expression cassettes in pCR01.
