# Supplementary material for: Establishing RNA virus resistance in plants by harnessing CRISPR immune system
Source: Plant Biotechnol J. 2018 Feb 14;16(8):1415–23. doi: 10.1111/pbi.12881 (PMC6041442; doi:10.1111/pbi.12881)
Supplement: Supplementary file 1 — Figure S1 Transient transformation of the sgRNA–FnCas9 system to develop CMV or TMV resistance in N. benthamiana. Figure S2 Structure of pCR01 and its variants used in the transient assay. Table S1 sgRNA target site IDs, sequences and oligonucleotides for cloning into pCR01. Table S2 Primers used in this study. Appendix S1 Sequence of the sgRNA and FnCas9 expression cassettes in pCR01. [file PBI-16-1415-s001.docx]

**Supplementary Figures**

**
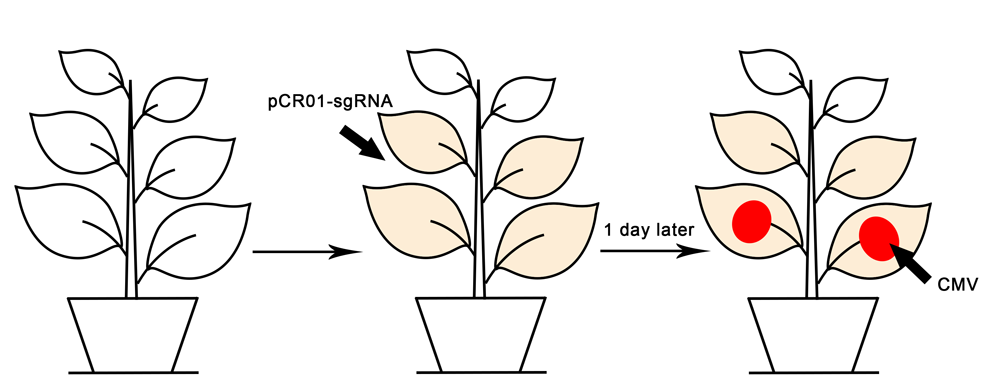
**

**Supplementary Figure 1** Transient transformation of the sgRNA-FnCas9 system to develop CMV or TMV resistance in *N. benthamiana*. Four-week-old *N. benthamiana* plants with six or seven true leaves were injected with *A. tumefaciens* strain EHA105 harboring the pCR01-sgRNA vector resuspended at a final OD_600_ of 1.0. One day later, CMV or TMV-GFP infectious clones were resuspended to a final OD_600_ of 0.6 and injected into the bottom two previously infected leaves.


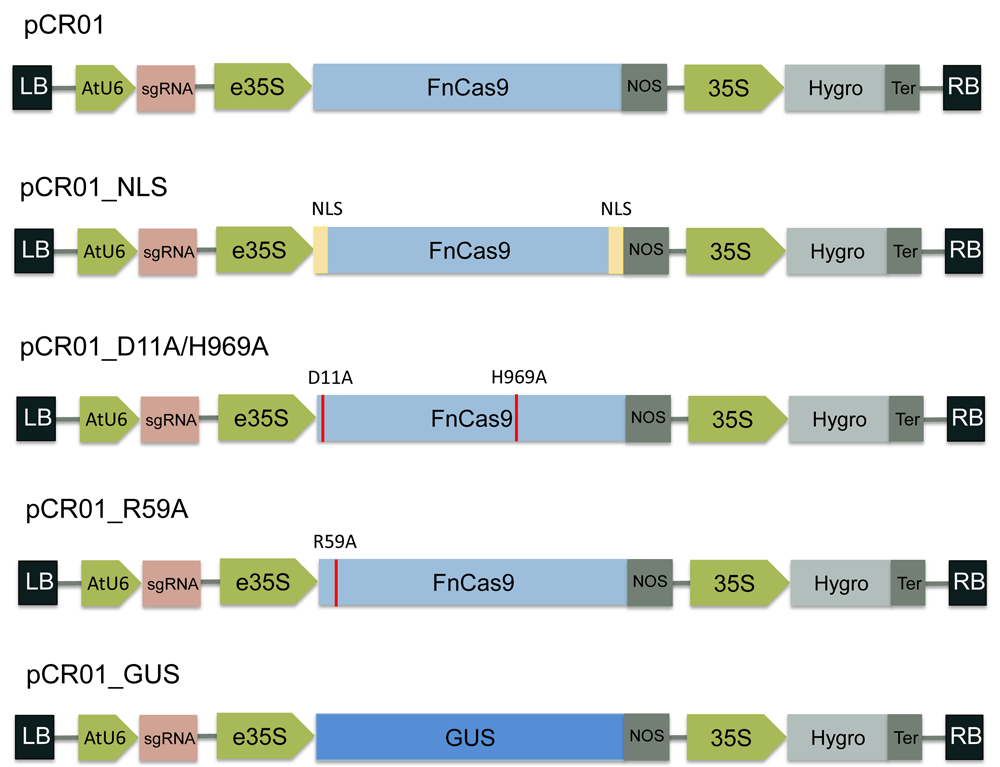


**Supplementary Figure 2** Structure of pCR01 and its variants used in the transient assay.

**Supplementary Tables**

**Supplementary Table 1.** sgRNA target site IDs, sequences and oligonucleotides for cloning into pCR01.

| **Target** | **sgRNA ID** | **CMV genome target sequence** | **Oligonucleotide sequence** |
| --- | --- | --- | --- |
| CMV  RNA1 | 1A | CCGCCCTCGTTGATAAGACAGC | F: gattGCTGTCTTATCAACGAGGG |
|  |  |  | R: cgagCCCTCGTTGATAAGACAGC |
|  | 1B | CCGGCTGCGGATTGCAAAGTAC | F: gattGTACTTTGCAATCCGCAGC |
|  |  |  | R: cgagGCTGCGGATTGCAAAGTAC |
|  | 1C | CCACTGACATGATCGTTACCGC | F: gattGCGGTAACGATCATGTCAG |
|  |  |  | R: cgagCTGACATGATCGTTACCGC |
|  | 1D | GGTGCGATGATGTTTGACGACC | F: gattGGTCGTCAAACATCATCGC |
|  |  |  | R: cgagGCGATGATGTTTGACGACC |
|  | 1E | GGTCCGAAGTCTGATGGAGAGC | F: gattGCTCTCCATCAGACTTCGG |
|  |  |  | R: cgagCCGAAGTCTGATGGAGAGC |
|  | 1F | CGACCGCCCTCGTTGATAAGAC | F: gattGTCTTATCAACGAGGGCGG |
|  |  |  | R: cgagCCGCCCTCGTTGATAAGAC |
|  | 1G | GAAGTCTGATGGAGAGCTTTAC | F: gattGTAAAGCTCTCCATCAGAC |
|  |  |  | R: cgagGTCTGATGGAGAGCTTTAC |
| CMV  RNA2 | 2A | CCAGCATTGCTATGGCCAGAGC | F: gattGCTCTGGCCATAGCAATGC |
|  |  |  | R: cgagGCATTGCTATGGCCAGAGC |
|  | 2B | CCATTTGTGACTCGACTCTGCC | F: gattGGCAGAGTCGAGTCACAAA |
|  |  |  | R: cgagTTTGTGACTCGACTCTGCC |
|  | 2C | CCTCTCGTTTAGAGTTATCGGC | F: gattGCCGATAACTCTAAACGAG |
|  |  |  | R: cgagCTCGTTTAGAGTTATCGGC |
|  | 2D | GGCCTGTCGTAATTACAGGCCC | F: gattGGGCCTGTAATTACGACAG |
|  |  |  | R: cgagCTGTCGTAATTACAGGCCC |
|  | 2E | GGAACTGACAGGGTCATGCCGC | F: gattGCGGCATGACCCTGTCAGT |
|  |  |  | R: cgagACTGACAGGGTCATGCCGC |
|  | 2F | CGAAGAAGCAGAGACGAAGGTC | F: gattGACCTTCGTCTCTGCTTCT |
|  |  |  | R: cgagAGAAGCAGAGACGAAGGTC |
|  | 2G | TCGGAACTGACAGGGTCATGCC | F: gattGGCATGACCCTGTCAGTTC |
|  |  |  | R: cgagGAACTGACAGGGTCATGCC |
| CMV  RNA3 | 3A | CCCGCTTTGGTGTCTTTCCAAC | F: gattGTTGGAAAGACACCAAAGC |
|  |  |  | R: cgagGCTTTGGTGTCTTTCCAAC |
|  | 3B | CCTCTTACTCCCTGTTGAGCCC | F: gattGGGCTCAACAGGGAGTAAG |
|  |  |  | R: cgagCTTACTCCCTGTTGAGCCC |
|  | 3C | CCTCCTCGGACTTATCCGTTGC | F: gattGCAACGGATAAGTCCGAGG |
|  |  |  | R: cgagCCTCGGACTTATCCGTTGC |
|  | 3D | GGCCCCTCGTTCCCGAAGTAAC | F: gattGTTACTTCGGGAACGAGGG |
|  |  |  | R: cgagCCCTCGTTCCCGAAGTAAC |
|  | 3E | GGACTTATCCGTTGCCGCCATC | F: gattGATGGCGGCAACGGATAAG |
|  |  |  | R: cgagCTTATCCGTTGCCGCCATC |
|  | 3F | CTCCTCGGACTTATCCGTTGCC | F: gattGGCAACGGATAAGTCCGAG |
|  |  |  | R: cgagCTCGGACTTATCCGTTGCC |
|  | 3G | GAGACGGACGAGCTAGTACTTC | F: gattGAAGTACTAGCTCGTCCGT |
|  |  |  | R: cgagACGGACGAGCTAGTACTTC |
| CMV  3’UTR | 3'UTR-A | GGAAGGACTTCGGTCCGTGTAC | F: gattGTACACGGACCGAAGTCCT |
|  |  |  | R: cgagAGGACTTCGGTCCGTGTAC |
|  | 3'UTR-B | TTCTTCGGAAGGACTTCGGTCC | F: gattGGACCGAAGTCCTTCCGAA |
|  |  |  | R: cgagTTCGGAAGGACTTCGGTCC |
| TMV | TA | CCTACTGAGGCGAATGTTGCGC | F: gattGCGCAACATTCGCCTCAGT |
|  |  |  | R: cgagACTGAGGCGAATGTTGCGC |
|  | TB | GGCAGAGTGTATGCCATTGCGC | F: gattGCGCAACATTCGCCTCAGT |
|  |  |  | R: cgagACTGAGGCGAATGTTGCGC |
|  | TC | AGGACGCGCAAGGAAGTCTTAG | F: gatt GCGCAACATTCGCCTCAGT |
|  |  |  | R: cgagACTGAGGCGAATGTTGCGC |

**Supplementary Table 2.** Primers used in this study.

| **Primer name** | **Primer sequence (5’-3’)** | **Purpose** |
| --- | --- | --- |
| rt CMV F  rt CMV R | GTGAACGGGTTGTCCATCCAG  GACCGAAGTCCTTCCGAAGAAAC | RT-qPCR for CMV viral RNA |
| rt TMV F  rt TMV R | CTTCCCGGCCTCTAATAGAGAG  GTAATGCCATGCGTCTTCCATTGC | RT-qPCR for TMV viral RNA |
| rt GFP F  rt GFP R | GATACGTGCAGGAGAGGACCATC  GCTTGTCGGCCATGATGTATAC | RT-qPCR for GFP expression |
| rt NbPP2A F  rt NbPP2A R | GACCCTGATGTTGATGTTCGCT  GAGGGATTTGAAGAGAGATTTC | RT-qPCR for PP2A of *N. benthamiana* |
| rt FnCAS9 F  rt FnCAS9 R | GATAAGGACACACAGCAGGCGAT  CTGCTCTGTCGCGAGCTTCAG | qPCR for FnCas9 expression |
| rt Actin8 F  rt Actin8 R | GAGACAACTTACAACTCGATC  CTGTGGACAATGCCTGGAC | qPCR for Actin8 of *Arabidopsis* |
| CMV S1 F  CMV S1 R | AAGAGCGTACGGTTCAATCCCT  GAGGTAATGTGTGACCCAACTTC | RT-PCR for CMV RNA1 |
| CMV S3 F  CMV S3 R | GACCGTGGGTCTTATTACGGTA  GAGCGCATCGTCTTTTGAATACAC | RT-PCR for CMV RNA3 |
| D11A F  D11A R | TCCCGATCGCCATTGCCCTGGGCGTGAAGAA  TTCTTCACGCCCAGGGCAATGGCGATCGGGA | Generate D11A mutation in FnCas9 |
| H969A F  H969A R | GAAGGAGGAGCTGGATGCCATCATTCCACGCT  AGCGTGGAATGATGGCATCCAGCTCCTCCTTC | Generate H969A mutation in FnCas9 |
| R59A F  R59A R | AGGACCGCGAGGCGCGCCCAGAGGAGGGGAAT  ATTCCCCTCCTCTGGGCGCGCCTCGCGGTCCT | Generate R59A mutation in FnCas9 |

**Supplementary Note**

Sequence of the sgRNA and FnCas9 expression cassettes in pCR01

**> AtU6-sgRNA**

CACGACGTTGTAAAACGACGGCCAGTGCCAAGCTTCATTCGGAGKTTTTGTATCTTGTTTCATAGTTTGTCCCAGGATTAGAATGATTAGGCATCGAACCTTCAAGAATTTGATTGAATAAAACATCTTCATTCTTAAGATATGAAGATAATCTTCAAAAGGCCCCTGGGAATCTGAAAGAAGAGAAGCAGGCCCATTTATATGGGAAAGAACAATAGTATTTCTTATATAGGCCCATTTAAGTTGAAAACAATCTTCAAAAGTCCCACATCGCTTAGATAAGAAAACGAAGCTGAGTTTATATACAGCTAGAGTCGAAGTAGTGATTGGAGACCTCGGACCTCGGTCTCGCTCGTAATTAATAAACCATGAAAGTATGGTTTATTAGATTGTTGAAGGCTAGTCCGTTATCAACTTGTTTTTTGTTTTAGAGCTAGAAATAGCAAGTTAAAATAAGGCTAGTCCGTAGCGCGTGCGCCAATTCTGCAGACAAATGGCCCCGGG

The AtU6 promoter and sgRNA are highlighted in green and magenta, respectively. The double BsaI sites for sgRNA cloning are underlined.

**>35S-FnCas9-Nos**

TGAGACTTTTCAACAAAGGGTGATATCCGGAAACCTCCTCGGATTCCATTGCCCAGCTATCTGTCACTTTATTGTGAAGATAGTGGAAAAGGAAGGTGGCTCCTACAAATGCCATCATTGCGATAAAGGAAAGGCCATCGTTGAAGATGCCTCTGCCGACAGTGGTCCCAAAGATGGACCCCCACCCACGAGGAGCATCGTGGAAAAAGAAGACGTTCCAACCACGTCTTCAAAGCAAGTGGATTGATGTGATATCTCCACTGACGTAAGGGATGACGCACAATCCCACTATCCTTCGCAAGACCCTTCCTCTATATAAGGAAGTTCATTTCATTTGGAGAGGACCTCGACCTCAACACAACATATACAAAACAAACGAATCTCAAGCAATCAAGCATTCTACTTCTATTGCAGCAATTTAAATCATTTCTTTTAAAGCAAAAGCAATTTTCTGAAAATTTTCACCATTTACGAACGATACCATGGACTACAAGGATCACGACGGCGATTACAAGGACCATGATATTGACTACAAGGACGATGACGATAAGGCCGCGGCCAACTTCAAGATCCTCCCGATCGCCATTGACCTGGGCGTGAAGAATACCGGCGTGTTCTCCGCGTTCTACCAGAAGGGCACAAGCCTGGAGAGGCTCGACAATAAGAACGGCAAGGTGTACGAGCTCTCCAAGGATAGCTACACCCTCCTGATGAACAATAGGACCGCGAGGCGCCACCAGAGGAGGGGAATTGACAGGAAGCAGCTGGTCAAGCGCCTGTTCAAGCTCATCTGGACCGAGCAGCTGAACCTCGAGTGGGATAAGGACACACAGCAGGCGATCTCATTCCTCTTCAATCGCCGGGGCTTCTCATTCATTACCGATGGCTACTCTCCAGAGTACCTCAACATCGTGCCTGAGCAGGTCAAGGCCATCCTGATGGACATTTTCGACGATTACAATGGCGAGGACGATCTCGATTCTTACCTGAAGCTCGCGACAGAGCAGGAGTCAAAGATCTCTGAGATCTACAACAAGCTCATGCAGAAGATTCTGGAGTTCAAGCTGATGAAGCTCTGCACCGACATCAAGGACGATAAGGTGTCAACCAAGACACTCAAGGAGATCACATCTTACGAGTTCGAGCTCCTGGCGGATTACCTCGCCAACTACTCAGAGTCTCTCAAGACCCAGAAGTTCTCATACACAGACAAGCAGGGCAATCTGAAGGAGCTCTCTTACTACCACCATGATAAGTACAACATCCAGGAGTTCCTCAAGCGCCACGCCACCATCAATGACCGGATTCTCGATACCCTCCTGACAGACGATCTGGACATCTGGAATTTCAACTTCGAGAAGTTCGATTTCGACAAGAACGAGGAGAAGCTCCAGAATCAGGAGGATAAGGACCACATTCAGGCCCATCTGCACCATTTCGTGTTCGCGGTCAATAAGATCAAGTCAGAGATGGCGTCTGGCGGCCGGCATAGGTCACAGTACTTCCAGGAGATCACCAACGTGCTCGACGAGAACAATCACCAGGAGGGCTACCTCAAGAACTTCTGCGAGAATCTGCATAACAAGAAGTACTCCAATCTGAGCGTGAAGAATCTGGTCAACCTCATTGGCAATCTCAGCAACCTGGAGCTCAAGCCACTGCGGAAGTACTTCAACGATAAGATCCACGCGAAGGCCGACCATTGGGATGAGCAGAAGTTCACCGAGACATACTGCCATTGGATTCTCGGCGAGTGGAGGGTGGGCGTCAAGGATCAGGACAAGAAGGACGGCGCCAAGTACTCCTACAAGGATCTCTGCAACGAGCTGAAGCAGAAGGTGACCAAGGCGGGCCTCGTCGACTTCCTCCTGGAGCTCGACCCGTGCCGCACAATTCCGCCATACCTCGACAACAATAACCGGAAGCCTCCGAAGTGCCAGAGCCTGATCCTCAATCCGAAGTTCCTGGATAATCAGTACCCAAACTGGCAGCAGTACCTGCAGGAGCTCAAGAAGCTGCAGTCCATCCAGAACTACCTCGACAGCTTCGAGACgGATCTCAAGGTGCTGAAGTCCAGCAAGGACCAGCCATACTTCGTCGAGTACAAGTCATCTAATCAGCAGATCGCCTCCGGCCAGCGGGATTACAAGGATCTCGACGCGCGCATCCTCCAGTTCATTTTCGACCGCGTGAAGGCCAGCGATGAGCTCCTGCTCAACGAGATCTACTTCCAGGCCAAGAAGCTCAAGCAGAAGGCGTCCAGCGAGCTCGAGAAGCTGGAGTCATCTAAGAAGCTGGACGAGGTCATCGCCAACTCACAGCTCTCTCAGATTCTGAAGTCCCAGCACACCAATGGCATCTTCGAGCAGGGCACATTCCTGCATCTCGTCTGCAAGTACTACAAGCAGAGGCAGAGGGCCAGGGACAGCAGGCTCTACATCATGCCAGAGTACCGCTACGATAAGAAGCTGCACAAGTACAATAACACCGGCCGCTTCGACGATGACAACCAGCTGCTCACATACTGCAATCATAAGCCGAGGCAGAAGCGCTACCAGCTGCTCAATGACCTCGCGGGAGTGCTGCAGGTgTCCCCAAATTTCCTCAAGGATAAGATCGGCTCAGATGACGATCTCTTCATTTCTAAGTGGCTGGTGGAGCACATCAGGGGCTTCAAGAAGGCCTGCGAGGACAGCCTGAAGATCCAGAAGGATAACAGGGGCCTGCTCAATCATAAGATCAACATTGCGCGCAATACCAAGGGCAAGTGCGAGAAGGAGATCTTCAACCTCATCTGCAAGATTGAGGGCTCCGAGGACAAGAAGGGCAATTACAAGCATGGACTGGCATACGAGCTGGGAGTCCTGCTCTTCGGAGAGCCTAACGAGGCGAGCAAGCCGGAGTTCGATCGCAAGATCAAGAAGTTCAATTCCATCTACAGCTTCGCGCAGATCCAGCAGATTGCGTTCGCCGAGCGGAAGGGCAATGCGAACACATGCGCGGTGTGCTCAGCCGACAATGCACATAGGATGCAGCAGATCAAGATTACAGAGCCTGTCGAGGACAATAAGGATAAGATCATTCTCTCTGCGAAGGCACAGAGGCTGCCAGCAATCCCTACCCGCATTGTGGATGGCGCGGTCAAGAAGATGGCGACAATTCTCGCCAAGAATATCGTGGACGATAATTGGCAGAACATTAAGCAGGTCCTCTCAGCCAAGCACCAGCTGCATATCCCTATCATTACCGAGTCTAATGCCTTCGAGTTCGAGCCAGCCCTGGCAGATGTGAAGGGCAAGTCCCTGAAGGATAGGCGCAAGAAGGCCCTCGAGCGGATTAGCCCGGAGAATATCTTCAAGGATAAGAATAACAGGATCAAGGAGTTCGCCAAGGGCATTTCAGCGTACTCTGGCGCCAACCTCACAGATGGCGACTTCGATGGCGCGAAGGAGGAGCTGGATCACATCATTCCACGCTCCCATAAGAAGTACGGCACCCTCAACGACGAGGCCAATCTGATTTGCGTCACACGGGGCGATAATAAGAACAAGGGCAACCGCATCTTCTGCCTCCGGGACCTGGCGGATAATTACAAGCTGAAGCAGTTCGAGACgACAGACGATCTCGAGATCGAGAAGAAGATTGCCGACACCATCTGGGATGCGAATAAGAAGGACTTCAAGTTCGGCAACTACAGGAGCTTCATTAATCTCACACCACAGGAGCAGAAGGCGTTCCGCCATGCACTGTTCCTCGCCGATGAGAACCCTATCAAGCAGGCCGTGATCCGCGCGATTAATAACCGCAATCGGACCTTCGTGAATGGCACACAGCGCTACTTCGCCGAGGTCCTCGCGAATAACATCTACCTCCGGGCCAAGAAGGAGAATCTCAACACCGACAAGATCTCATTCGATTACTTCGGCATCCCAACAATTGGCAACGGCCGGGGCATCGCCGAGATTAGGCAGCTGTACGAGAAGGTCGACTCTGATATCCAGGCGTACGCCAAGGGCGACAAGCCTCAGGCGTCCTACAGCCACCTCATTGATGCGATGCTGGCCTTCTGCATCGCGGCCGACGAGCATAGGAACGATGGCTCAATTGGCCTCGAGATCGACAAGAACTACTCTCTCTACCCGCTGGATAAGAATACCGGCGAGGTGTTCACAAAGGACATCTTCTCCCAGATCAAGATTACCGACAACGAGTTCAGCGATAAGAAGCTGGTCCGCAAGAAGGCCATTGAGGGCTTCAACACCCACAGGCAGATGACACGCGATGGCATCTACGCGGAGAATTACCTCCCAATCCTGATTCATAAGGAGCTCAACGAGGTGAGGAAGGGCTACACATGGAAGAATTCCGAGGAGATCAAGATTTTCAAGGGCAAGAAGTACGACATCCAGCAGCTCAATAACCTGGTGTACTGCCTCAAGTTCGTCGACAAGCCTATCTCAATTGATATCCAGATTTCTACCCTCGAGGAGCTGCGCAACATCCTGACCACAAATAACATTGCGGCCACCGCCGAGTACTACTACATTAATCTCAAGACACAGAAGCTGCACGAGTACTACATCGAGAATTACAACACAGCCCTCGGCTACAAGAAGTACAGCAAGGAGATGGAGTTCCTCAGGTCCCTGGCGTACCGGAGCGAGAGGGTGAAGATCAAGTCCATTGACGATGTGAAGCAGGTCCTCGACAAGGACAGCAACTTCATCATTGGCAAGATTACCCTGCCTTTCAAGAAGGAGTGGCAGCGCCTCTACCGGGAGTGGCAGAACACCACAATCAAGGACGATTACGAGTTCCTCAAGTCCTTCTTCAATGTGAAGAGCATTACAAAGCTGCATAAGAAGGTCCGCAAGGACTTCTCCCTCCCGATCAGCACCAACGAGGGCAAGTTCCTGGTGAAGCGCAAGACCTGGGATAATAACTTCATCTACCAGATTCTCAATGACTCAGATTCTAGGGCCGACGGCACCAAGCCGTTCATTCCAGCGTTCGATATCTCCAAGAACGAGATTGTCGAGGCCATCATTGACTCCTTCACAAGCAAGAACATCTTCTGGCTCCCTAAGAATATTGAGCTGCAGAAGGTCGACAATAAGAACATCTTCGCCATTGATACCTCCAAGTGGTTCGAGGTCGAGACACCTAGCGACCTCCGCGATATCGGCATTGCGACCATTCAGTACAAGATCGACAATAACTCACGGCCGAAGGTGAGGGTCAAGCTGGATTACGTGATCGACGATGACTCTAAGATTAACTACTTCATGAATCACTCACTGCTCAAGTCTAGGTACCCGGATAAGGTCCTCGAGATCCTGAAGCAGTCAACCATCATTGAGTTCGAGTCCAGCGGCTTCAACAAGACAATTAAGGAGATGCTCGGCATGAAGCTGGCCGGCATCTACAATGAGACgTCTAATAACTGAGGATCCTGATTGATCGATAGAGCTCGAATTTCCCCGATCGTTCAAACATTTGGCAATAAAGTTTCTTAAGATTGAATCCTGTTGCCGGTCTTGCGATGATTATCATATAATTTCTGTTGAATTACGTTAAGCATGTAATAATTAACATGTAATGCATGACGTTATTTATGAGATGGGTTTTTATGATTAGAGTCCCGCAATTATACATTTAATACGCGATAGAAAACAAAATATAGCGCGCAAACTAGGATAAATTATCGCGCGCGGTGTCATCTATGTTACTAGATCGGGAATTC

The 35S promoter, FnCas9, Nos terminator and 3*FLAG sequences are highlighted in the corresponding colors.
